# Mortality in men with castration‐resistant prostate cancer—A long‐term follow‐up of a population‐based real‐world cohort

**DOI:** 10.1002/bco2.116

**Published:** 2021-10-10

**Authors:** Yashar Khoshkar, Marcus Westerberg, Jan Adolfsson, Anna Bill‐Axelson, Henrik Olsson, Martin Eklund, Olof Akre, Hans Garmo, Markus Aly

**Affiliations:** ^1^ Department of Molecular Medicine and Surgery Karolinska Institute Stockholm Sweden; ^2^ Department of Mathematics Uppsala University Uppsala Sweden; ^3^ Department of Surgical Sciences Uppsala University Uppsala Sweden; ^4^ Department of Clinical Science, Intervention and Technology Karolinska Institute Stockholm Sweden; ^5^ Department of Medical Epidemiology and Biostatistics Karolinska Institute Stockholm Sweden; ^6^ Prostate Cancer Flow, Patient Area Pelvic Cancer Karolinska Sjukhuset Solna Stockholm Sweden; ^7^ Translational Oncology & Urology Research (TOUR), School of Cancer and Pharmaceutical Sciences King's College London London UK; ^8^ Regional Cancer Centre, Division of Central Sweden Uppsala Sweden

**Keywords:** castration resistant prostate cancer, mortality, mortality in castration resistant prostate cancer, natural history cohort, PSA doubling time, PSA at time of CRPC, real‐world cohort

## Abstract

**Objectives:**

The objective of this study is to find clinical variables that predict the prognosis for men with castration‐resistant prostate cancer (CRPC) in a Swedish real‐life CRPC cohort, including a risk group classification to clarify the risk of succumbing to prostate cancer. This is a natural history cohort representing the premodern drug era before the introduction of novel hormonal drug therapies.

**Methods:**

PSA tests from the clinical chemistry laboratories serving health care in six regions of Sweden were retrieved and cross‐linked to the National Prostate Cancer Registry (NPCR) to identify men with a prostate cancer diagnosis. Through further cross‐linking with data sources at the Swedish Board of Health and Welfare, we retrieved other relevant information such as prescribed drugs, hospitalizations, and cause of death. Men entered the CRPC cohort at the first date of doubling of their PSA nadir value with the last value being >2 ng/ml, or an absolute increase of >5 ng/ml or more, whilst on 3 months of medical castration or if they had been surgically castrated (*n* = 4098). By combining the two variables with the largest C‐statistics, “PSA at time of CRPC” and “PSA doubling time,” a risk group classification was created.

**Results:**

PSA‐DT and PSA at date of CRPC are the strongest variables associated with PC specific survival. At the end of follow‐up, the proportion of men who died due to PC was 57%, 71%, 81%, 86%, and 89% for risk categories one through five, respectively. The median overall survival in our cohort of men with CRPC was 1.86 years (95% CI: 1.79–1.97).

**Conclusion:**

For a man with castration‐resistant prostate cancer, there is a high probability that this will be the main cause contributing to his death. However, there is a significant difference in mortality that varies in relation to tumor burden assessed as PSA doubling time and PSA at time of CRCP. This information could be used in a clinical setting when deciding when to treat more or less aggressively once entering the CRPC phase of the disease.

## 1 INTRODUCTION

When prostate cancer (PC) treated with castration evolves into a castration‐resistant phase, it was previously considered as the point where survival could not be prolonged. The introduction of docetaxel in 2004 resulted in improved survival in patients with metastasized castration‐resistant PC (mCRPC).^1^, ^2^ Thereafter, chemotherapy has been initiated earlier in the disease trajectory,^3^, ^4^ with a considerable survival benefit with docetaxel treatment added to ADT in metastatic hormone‐sensitive PC (mHSPC). Some of the latest treatment options improving overall survival in patients with CRPC, both in a predocetaxel and postdocetaxel treatment setting, are abiraterone acetate,^5^, ^6^ enzalutamide,^7^, ^8^ and darolutamide.^9^

There is limited follow‐up data on the mortality outcome of the castration‐resistant phase of PC outside clinical trials. We have identified and followed a population‐based cohort of men (*n* = 4098) with CRPC, with the aim to identify clinical variables that may predict the prognosis for these patients. Second, we aim to describe the proportions of CRPC patients that succumb to PC compared with other causes. This study also aims to describe the natural course of a real world CRPC cohort, in a premodern setting before the introduction of novel treatments such as abiraterone acetate, enzalutamide, and darolutamide.

## 2 MATERIAL

### 2.1 Data sources

All PSA tests along with national patient identification number of the men from the clinical chemistry laboratories serving health care in six regions of Sweden were retrieved. The personal identification number were linked to the National Prostate Cancer Register (NPCR) to identify men with a PC diagnosis. From the NPCR, we retrieved the staging information based on the TNM (tumour, node, metastasis) classification from the national prostate cancer registry, Gleason grading, WHO grading, diagnostic work‐up, and primary treatment.

Through a second record linking with data sources at the Swedish Board of Health and Welfare, we retrieved information on treatment history, comorbidity, and cause of death. We ascertained ADT through data in the Swedish Prescribed Drug Register, retrieved information on comorbidity from the National Patient Register and the National Cancer Register. Cause of death was retrieved from the National Cause of Death Register. Socioeconomic data including educational level and civil status were retrieved from the Longitudinal Integration Database for Health Insurance and Labor Market Studies (LISA) held at Statistics Sweden. The data record linkage described above was done separately for the Stockholm and Uppsala regions.

### 2.2 Study population

We obtained all PSA measurements during the period 2005–2014 from the Uppsala/Örebro PSA cohort (UPSAC) including five counties in the region of Uppsala/Örebro.^10^ Similarly, we retrieved data for the Stockholm region from the STHLM‐0 cohort^11^ during the period 2003–2017. These two cohorts represent almost 40% of the population in Sweden.^12^ Men were included if they are (1) registered in the NPCR, (2) treated with GnRH for the first time after 2006‐01‐01, and (3) fulfilling the definition of CRPC as defined in Section 2.3.3.

Follow‐up ended at death or 2014‐12‐31 for men in the Uppsala region and 2016‐12‐31 for men in the Stockholm region. Men migrating out of the corresponding region to another region or another country were censored at the time of migration.

**FIGURE 1:**
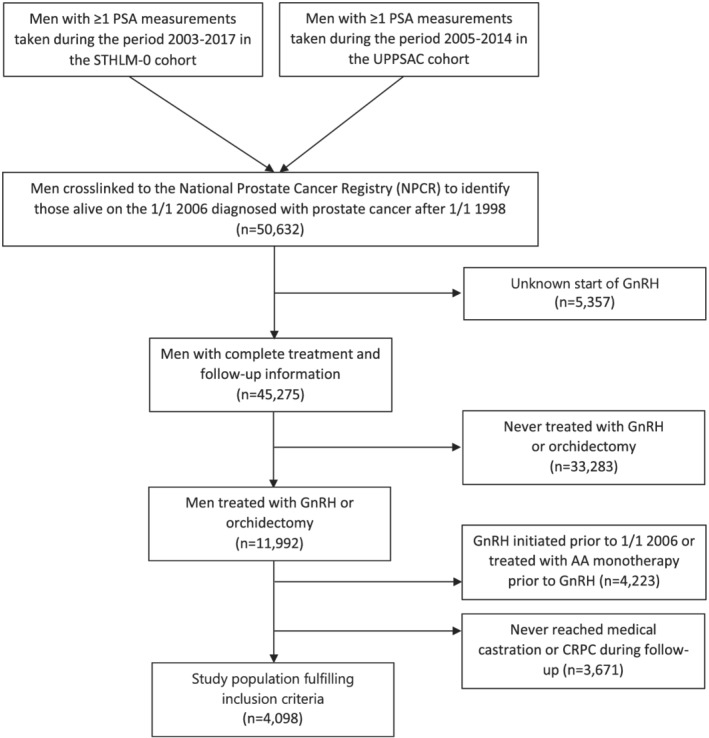
Consort diagram presenting the selection of CRPC men

We differed between CRPC patients treated with neoadjuvant ADT prior to radiotherapy from CRPC patients treated with ADT due to disease progression and ADT as primary treatment, as described in Hemelrijck's publication for Prostate Cancer data Base Sweden (PCBaSe).^13^ Figure 1 presents the patient selection flow chart.

### 2.3 Study definitions and variables

#### 2.3.1 Medical castration

Medical castration was reached when patients had been treated with ADT for at least 3 months within a total period of 6 months according to records in the Swedish National Prescribed Drug Registry.

#### 2.3.2 Surgical castration

These patients have a record of surgical castration in the Swedish National Prostate Cancer Registry. The hospital admission date related to the surgical castration was used as an estimate of the castration date.

#### 2.3.3 Castration‐resistant prostate cancer

The date of CRPC is based on an increase in PSA (first date of doubling of nadir PSA value with the last value being greater than 2 ng/ml, or an absolute increase of 5 ng/ml or more) despite medical or surgical castration.

#### 2.3.4 Primary versus secondary ADT

Primary ADT is defined as ADT initiated as first treatment for PC. Secondary ADT is defined as ADT initiated following recurrence of PC that occurs after curative intent prostatectomy or radiotherapy. This group also includes patients on deferred ADT.

#### 2.3.5 M status

M1/M0 status is based upon PC diagnosis.

#### 2.3.6 Risk group classification

We decided to apply measures of PSA‐kinetics combining the two variables with the largest C‐statistics (see Section 2.4) that remained mainly unchanged in a full multivariable model, which were “PSA at time of CRPC” and “PSA‐DT.” Interaction effects between the log of PSA at date of CRCP against log PSA‐DT were investigated in a Cox model by the use of a restricted two‐dimensional cubic spline and the *gam*‐function in the R‐package *mgcv*.^14^, ^15^ The equation for the stratification into risk groups 1–5 is as follows: Score = log (PSA at CRPC) − 1.40 * log (PSA‐DT).

If score ≤−6.09 = Risk group 1.

If >−6.09 but ≤−4.73 = Risk group 2.

If>−4.73 but ≤−3.34 = Risk group 3.

If>−3.34 but ≤ − 1.58 = Risk group 4.

If>−1.58 = Risk group 5.

As we knew that our M‐stage variable was only reflecting M‐stage at PC diagnosis, this study was not intended to create a complete validated risk score of CRPC. Therefore, no other covariates were used when creating our score. CRPC men having low risk characteristics with low PSA at the date of CRPC and long PSA‐DT belong to risk group 1, whereas these values are vice versa for risk group 5 patients (Figure 3).

### 2.4 Statistical analysis

**FIGURE 2:**
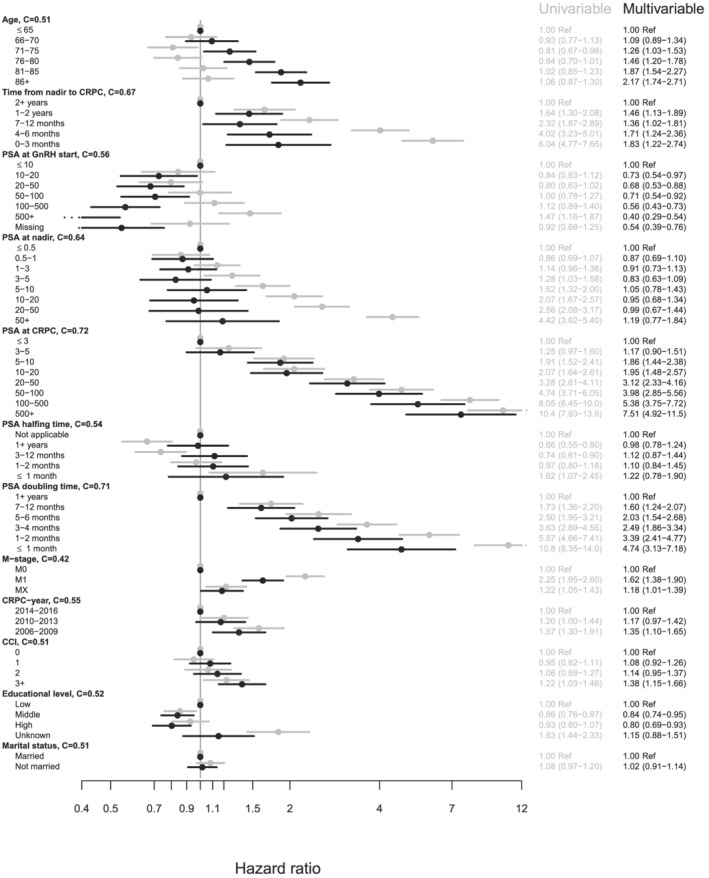
Hazard ratios for PC death in univariable and multivariable Cox‐regression models in men with primary ADT

Univariable and multivariable Cox proportional hazards regression was performed for overall mortality and PC specific mortality.^16^ Cumulative incidence of death was computed considering death from PC and other causes as competing risks, censoring for end of follow‐up or date of move from the region. Harrell's C‐index, also known as the concordance index, is a goodness‐of‐fit measure for models that produce risk scores.^17^ C‐index was calculated for each variable in Figure 2 to validate those being most representative for predicting mortality.

## 3 RESULTS

### 3.1 Baseline characteristics

A total of 4098 patients were eligible for analysis (Table 1), of which 1117 with metastasis (M1), 1340 without metastases (M0), and 1641 with unknown status (Mx) at the time of PC diagnosis. In all, 551 patients underwent primary treatment with curative intention and 2574 patients were treated with primary ADT, of which 257 underwent surgical castration. Descriptive data for the whole cohort stratified on primary and nonprimary castration treatment is given in Table 1.

**TABLE 1:** Baseline characteristics for men with castration‐resistant prostate cancer

	Primary GnRH/orchiectomy (*n* = 2574)	Nonprimary GnRH/orchiectomy (*n* = 1524)	All (*n* = 4098)
Age, *n* (%)			
≤65	301 (12)	160 (10)	461 (11)
66–70	349 (14)	202 (13)	551 (13)
71–75	432 (17)	272 (18)	704 (17)
76–80	515 (20)	309 (20)	824 (20)
81–85	541 (21)	329 (22)	870 (21)
86+	436 (17)	252 (17)	688 (17)
Time_from_Nadir, *n* (%)			
0–3 months	303 (12)	212 (14)	515 (13)
4–6 months	580 (23)	382 (25)	962 (23)
7–12 months	748 (29)	451 (30)	1199 (29)
1–2 years	563 (22)	288 (19)	851 (21)
2+ years	380 (15)	191 (13)	571 (14)
Time_from_GnRH, *n* (%)			
0–6 months	271 (11)	263 (17)	534 (13)
7–12 months	726 (28)	476 (31)	1202 (29)
1–2 years	783 (30)	414 (27)	1197 (29)
2+ years	794 (31)	371 (24)	1165 (28)
Time_from_DX, *n* (%)			
0–6 months	140 (5)		140 (3)
7–12 months	709 (28)	5 (0)	714 (17)
1–2 years	839 (33)	85 (6)	924 (23)
2–4 years	628 (24)	374 (25)	1002 (24)
4+ years	258 (10)	1060 (70)	1318 (32)
CRPC_year, *n* (%)			
2006–2009	719 (28)	352 (23)	1071 (26)
2010–2013	1305 (51)	761 (50)	2066 (50)
2014–2017	550 (21)	411 (27)	961 (23)
PSA at GnRH start, *n* (%)			
≤10 ng/ml	188 (7)	380 (25)	568 (14)
10–20 ng/ml	232 (9)	318 (21)	550 (13)
20–50 ng/ml	509 (20)	367 (24)	876 (21)
50–100 ng/ml	435 (17)	204 (13)	639 (16)
100–500 ng/ml	694 (27)	170 (11)	864 (21)
500+ ng/ml	385 (15)	38 (2)	423 (10)
Missing	131 (5)	47 (3)	178 (4)
PSA at Nadir, *n* (%)			
≤0.5 ng/ml	512 (20)	241 (16)	753 (18)
0.5–1 ng/ml	331 (13)	136 (9)	467 (11)
1–3 ng/ml	591 (23)	293 (19)	884 (22)
3–5 ng/ml	258 (10)	131 (9)	389 (9)
5–10 ng/ml	252 (10)	195 (13)	447 (11)
10–20 ng/ml	202 (8)	185 (12)	387 (9)
20–50 ng/ml	200 (8)	171 (11)	371 (9)
50+ ng/ml	228 (9)	172 (11)	400 (10)
PSA at CRPC, *n* (%)			
≤3 ng/ml	403 (16)	198 (13)	601 (15)
3–5 ng/ml	341 (13)	155 (10)	496 (12)
5–10 ng/ml	415 (16)	221 (15)	636 (16)
10–20 ng/ml	367 (14)	225 (15)	592 (14)
20–50 ng/ml	352 (14)	298 (20)	650 (16)
50–100 ng/ml	221 (9)	156 (10)	377 (9)
100–500 ng/ml	342 (13)	189 (12)	531 (13)
500+ ng/ml	133 (5)	82 (5)	215 (5)
PSA halving time, *n* (%)			
≤1 month	44 (2)	201 (13)	245 (6)
1–2 months	641 (25)	191 (13)	832 (20)
3–12 months	750 (29)	243 (16)	993 (24)
1+ years	907 (35)	566 (37)	1473 (36)
Not applicable	232 (9)	323 (21)	555 (14)
PSA doubling time, *n* (%)			
≤1 month	183 (7)	74 (5)	257 (6)
1–2 months	489 (19)	259 (17)	748 (18)
3–4 months	637 (25)	387 (25)	1024 (25)
5–6 months	353 (14)	266 (17)	619 (15)
7–12 months	540 (21)	328 (22)	868 (21)
1+ years	372 (14)	210 (14)	582 (14)
Treatment history *n* (%)			
GnRH	2317 (90)		2317 (57)
ORCH	257 (10)		257 (6)
Deferred treatment		973 (64)	973 (24)
Curative treatment		551 (36)	551 (13)
CCI at CRPC, *n* (%)			
0	1601 (62)	915 (60)	2516 (61)
1	413 (16)	254 (17)	667 (16)
2	273 (11)	155 (10)	428 (10)
3+	287 (11)	200 (13)	487 (12)
Educational level, *n* (%)			
Low	998 (39)	512 (34)	1510 (37)
Middle	924 (36)	566 (37)	1490 (36)
High	535 (21)	403 (26)	938 (23)
Unknown	117 (5)	43 (3)	160 (4)
Civil status, *n* (%)			
Married	1489 (58)	974 (64)	2463 (60)
Not married	1085 (42)	550 (36)	1635 (40)
T‐stage, *n* (%)			
T1	284 (11)	434 (28)	718 (18)
T2	550 (21)	548 (36)	1098 (27)
T3	1273 (49)	468 (31)	1741 (42)
T4	384 (15)	28 (2)	412 (10)
Missing	83 (3)	46 (3)	129 (3)
N‐stage, *n* (%)			
N0	127 (5)	178 (12)	305 (7)
N1	211 (8)	65 (4)	276 (7)
NX	2236 (87)	1281 (84)	3517 (86)
M‐stage, *n* (%)			
M0	652 (25)	688 (45)	1340 (33)
M1	1060 (41)	57 (4)	1117 (27)
MX	862 (33)	779 (51)	1641 (40)
Gleason grade, *n* (%)			
GGG1	84 (3)	339 (22)	423 (10)
GGG2	205 (8)	265 (17)	470 (11)
GGG3	362 (14)	261 (17)	623 (15)
GGG4	524 (20)	239 (16)	763 (19)
GGG5	948 (37)	182 (12)	1130 (28)
Only WHO grade	86 (3)	61 (4)	147 (4)
Missing	365 (14)	177 (12)	542 (13)
PSA at diagnosis, *n* (%)			
≤10 ng/ml	167 (6)	457 (30)	624 (15)
10–20 ng/ml	252 (10)	396 (26)	648 (16)
20–50 ng/ml	539 (21)	372 (24)	911 (22)
50–100 ng/ml	454 (18)	154 (10)	608 (15)
100–500 ng/ml	707 (27)	90 (6)	797 (19)
500+	405 (16)	11 (1)	416 (10)
Missing data	50 (2)	44 (3)	94 (2)

Abbreviations: CCI, Charlson Comorbidity Index; GGG, Gleason grade group; ORCH, orchidectomy.

### 3.2 Risk factors

The variables with the most significant c‐statistics for PC mortality were PSA at date of CRPC and PSA‐DT (Figure 2). Men with a PSA DT of <1 month have an HR for PC death of 4.76 (95% CI: 3.14–7.22) in the adjusted model compared with the men with a 1+ year DT. The shorter the DT, the higher the HR for PC death, resulting in a progressive risk increase. Men with a PSA of >500 ng/ml at the date of CRPC have an HR for PC death of 7.39 (95% CI: 4.84–11.30) compared with men with a PSA level of ≤3 ng/ml at the date of CRPC in the adjusted model. The increase in HR for PC death for the compared groups is in concordance with the level of the PSA.

Known bone metastasis at the time of diagnosis showed an adjusted HR for PC death of 1.62 (95% CI: 1.38–1.90) compared with M0 patients. Men diagnosed with CRPC year 2006–2009 have an adjusted HR for PC death of 1.35 (95% CI: 1.10–1.65) compared with men diagnosed in 2014–2016. Time from nadir to CRPC and PSA level at nadir affects the hazard ratios for PC death progressively in the unadjusted models, but neither of these variables has an influence on the HRs in the adjusted analysis. Neither PSA at GnRH start nor PSA halving time influence the HR for adjusted or unadjusted models.

### 3.3 Survival in the cohort

Median OS in the entire CRPC cohort of 4098 patients regardless of M‐status is 1.86 years (95% CI: 1.79–1.97). For CRPC patients treated with primary ADT, median OS is 1.71 years (95% CI: 1.62–1.82), and for the secondary ADT group, median OS was 2.10 years (95% CI: 1.99–2.23).

### 3.4 Survival of risk groups

**FIGURE 3:**
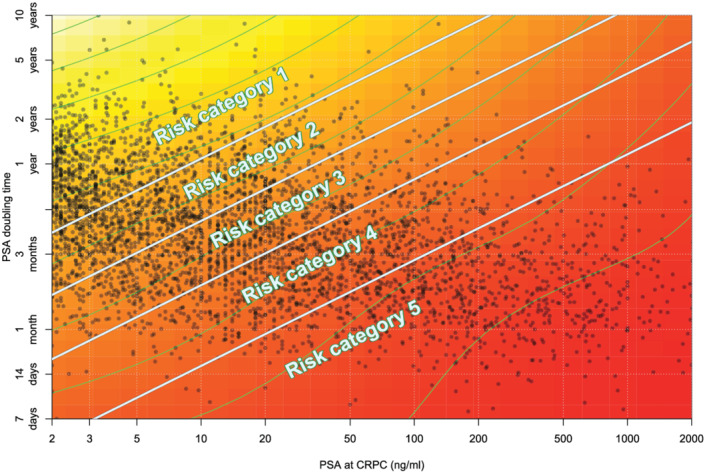
Heat map of risk of PC death based on PSA at date of CRPC and PSA‐DT illustrating the combined effect of these two variables. Dark red indicates a higher risk whilst a yellow color indicates a lower risk

Figure 3 describes that lower risk group classification results in lower mortality in CRPC. Men in risk group 1 have a median OS of 3.74 years (95% CI: 3.52–4.09), whereas men in risk group 5 have a median OS of 0.62 years (95% CI: 0.54–0.71), regardless of primary or secondary ADT. At the end of the follow‐up, the proportion death from PC was 57%, 71%, 81%, 86%, and 89% for risk categories 1–5.

## 4 DISCUSSION

### 4.1 Risk factors

Combining PSA at date of CRPC and PSA‐DT creates a clearer assessment of the aggressiveness of the CRPC disease. PSA‐DT is a well‐used study variable in both clinical and epidemiological studies already published.^18^, ^19^ Moreira et al. found in two retrospective analyses in men with nmCRPC (*n* = 458) and mCRPC (*n* = 205) that shorter PSA‐DT and higher PSA at the date of CRPC diagnosis were significantly associated with shorter time to metastasis and shorter OS.^20^, ^21^ Other biomarkers such as circulating tumor cells (CTCs) have shown to be superior to PSA kinetics in predicting the outcome for CRPC patients,^22^ but such information is lacking in the present data.

The absence of M‐status at time of CRPC is a shortcoming of this study. Our findings indicate that M‐status at PC diagnosis is less predictive in comparison with these two PSA variables. It is possible that an M‐stage at date of CRPC is more predictive and that a risk categorization including such an M‐stage would perform better in predicting PC death. We also noted a slightly better prognosis for patients being diagnosed with CRPC during later years (2014–2016) in this cohort, which is believed to be a result of a successively higher use of docetaxel during this follow‐up. It might also be an effect of changing selection mechanisms to AA monotherapy (men excluded from our study) during the inclusion period of our study.

### 4.2 Survival of the cohort

The OS in our real‐life cohort of men with CRPC is 1.86 years (95% CI: 1.79–1.97), which is in parity with a Dutch real‐life cohort of CRPC patients diagnosed in 2010–2012 whose OS is 2 years.^23^ This is in contrast to a clinical trial in which the OS is more than 40 months in patients with mCRPC,^24^ indicating that real‐life cohorts have a broader patient selection. Few other real‐world analyses on survival in CRPC men in premodern cohorts have been presented to our knowledge and include a much lower number of patients.^25^ A European multinational real‐world study with 481 patients from the postmodern drug era had time to treatment failure as their primary outcome but did not include overall survival analyses.^26^ Another real‐world study on a CRPC population has instead primarily focused on treatment patterns^27^ and has not analyzed cause of death in contrast to this study. A small postmodern CRPC cohort compared survival in men on the first‐ to fourth‐line enzalutamide treatment, but median OS times were not reached because of a short follow‐up time of 7.8 months.^28^

### 4.3 Survival of risk groups

Men in risk group 1 have a median OS of 3.74 years (95% CI: 3.52–4.09), whereas men in risk group 5 have a median OS of 0.62 years (95% CI: 0.54–0.71); 43% of the patients in risk group 1 appear to progress slow enough to succumb to diseases other than PC, indicating that the entity of CRPC and risk of dying differs among these men. Patients in risk group 1 can have a PSA‐DT being as low as 4 months or a PSA at date of CRPC being up to 200 as illustrated in Figure 3, which is paradox as these values indicate a more aggressive disease course. A suggestion of a clinical implication may be to additionally treat patients with a more aggressive disease course like those in risk group 5 with no delay and with less regard to comorbidities that otherwise may affect the decision of additional treatment. Patients belonging to risk groups 2–4 may be treated as recommended in guidelines. Risk group 1 patients may be subject of deferred treatment due to the remarkably good prognosis of these patients both with regard to longer overall survival and thereby the chance of succumbing to other reasons.

### 4.4 Study limitations

Hospital‐administered drugs such as docetaxel and other chemotherapies are not captured in the Prescribed Drug Register and therefore not recorded in this study. According to a Swedish real‐world cohort study published 2013 by Lissbrandt et al., 21% of the CRPC patients received chemotherapy.^29^ Thus, there is reason to believe that most of the patients in our cohort have only received ADT.

A relatively low number of patients had prior curative treatment (*n* = 551) that is because the follow up is not long enough to capture a larger number of these patients as men during this time period go through several phases of the PC disease trajectory. First, they have to be diagnosed with PC to then be curatively treated, have a PSA relapse that may or may not be treated with salvage radiation therapy or surgical therapy, and thereafter have second PSA relapse and then be put on GnRH in order to develop CRPC to be included in the studied cohort. If we would redo this analysis in 10 years, the numbers would look different, with more men having undergone curative treatment.

Men on antiandrogen monotherapy with bicalutamide were excluded from this study resulting in a cohort being not completely representative compared with GNRH cohorts in non‐Nordic countries. This treatment strategy was primarily implemented during 2006–2009 when the patent of bicalutamide expired meaning our cohort has a different selection depending on time periods, which is a potential limitation of this study.

**FIGURE 4:**
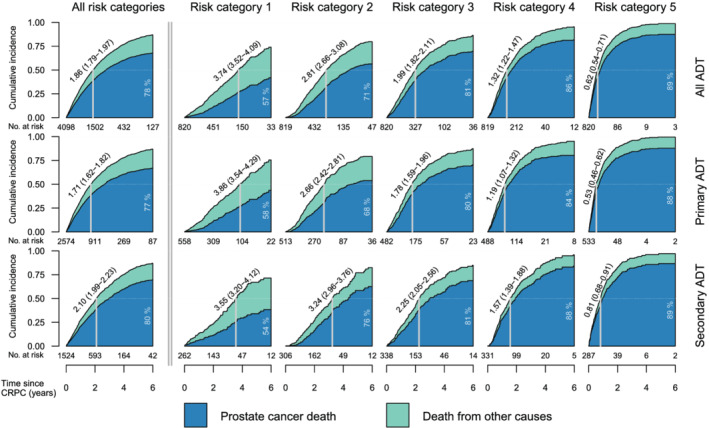
Stacked cumulative incidence of PC death and death of other causes in men with primary ADT and secondary ADT by the defined CRPC‐risk categories in Figure 3

M‐status of our cohort is not specified for the patients when entering the CRPC phase of their disease, creating an uncertainty in the heat map (Figure 3) and the survival model (Figure 4) as nmCRPC patients have longer expected survival than patients with mCRPC. PSA level at baseline is probably a surrogate for the tumor volume, and because most of our patients have a PSA at time of CRPC being <20, the cohort is believed to mainly include nmCRPC men with higher OS than mCRPC men. Likewise, there is no information of visceral metastasis for the patients of this study, which is highly relevant as these men are expected to have an even worse prognosis than for mCRPC patients with skeletal metastasis only.^30^ Despite the lack of M‐status at the time of CRPC, all patients that nowadays are diagnosed with CRPC are likely to be in one of these risk groups and the use of novel antiandrogens are only indicated once entering the CRPC phase with or without metastasis. Furthermore, the observed survival times may not be generalizable to contemporary patients as the study period is outside the era of new therapies in advanced PC resulting in a premodern CRPC cohort. Despite these issues, we believe that this study adds information that might be useful when deciding when and who to treat with novel AA or with chemotherapy. In a future study, we also aim to investigate whether or not these data are valid in separate cohorts.

The algorithm used to identify men with CRPC, using increase in PSA whilst on hormonal treatment, and without the use of testosterone may lead to minor misclassification and loss of some patients where clinical action was taken prior to an increase in PSA, which led to the inclusion in this cohort.

### 4.5 Study strengths

We performed sensitivity analysis in which PSA measurements taken after a long period without GnRH were ignored. The result did not change as could be expected given the high adherence presented in Gincy George's publication on long‐term medical adherence to GNRH agonist treatment in men with PC in Sweden.^31^

The source population of this study includes almost 40% of the Swedish population and a total of 4098 CRPC patients, which is one of the largest CRPC cohorts to our knowledge. The length of the follow up and the high‐quality data in our population‐based registers^32^ are also major strengths of this study. It is a real‐life study including patients with a larger burden of comorbidity and higher age, thus being more representative for clinical purpose.

Our aim with this study was to explore if and how clinically commonly used variables were associated with the risk of mortality in this group of patients. The findings of this study are mainly of hypothesis generating nature, and our aim is to further explore the results in future separate cohorts to eventually create a clinically useful tool to predict the outcome of patients with CRPC.

## 5 CONCLUSION

This CRPC cohort that for the most part likely consists of nmCRPC patients indicates that men with risk group 1 characteristics run a 43% chance of not dying of their disease. Predictive variables like PSA‐DT and PSA at the date of CRPC can therefore be of value to identify patients belonging to low‐ or high‐risk groups, respectively, benefiting from either a slightly less aggressive treatment approach or initiation of treatment with no delay and less regard to comorbidities.

## AUTHOR CONTRIBUTION

Yashar Khoshkar, Hans Garmo, Marcus Westerberg, Markus Aly, Jan Adolfsson, Olof Akre ‐ conception and design. Hans Garmo, Marcus Westerberg, Markus Aly, Martin ‐ acquisition of data Markus Aly, Yashar Khoshkar, Marcus Westerberg, Hans Garmo, Olof Akre, Jan Adolfson, Anna Bill‐Axelsson ‐ Analysis and interpretation of data. Yashar Khoskhar, Markus Aly, Marcus Westerberg, Hans Garmo ‐ Drafting of the manuscript Markus Aly, Hans Garmo, Olof Akre, Anna Bill‐Axelsson ‐ critical revision of the manuscript for imporant intellectual content. Hans Garmo, Marcus Westerberg, Markus Aly, Martin Eklund, Henrik Olsson ‐ Statistical analyses. Markus Aly, Jan Adolfson, Marcus Westerberg. Anna Bill‐Axelsson, Olof Akre ‐ Obtaining funding. Hans Garmo ‐ Administrative, technical, or material support Markus Aly, Hans Garmo, Olof Akre – Supervision.
